# Acute direct inguinal hernia resulting from blunt abdominal trauma: Case Report

**DOI:** 10.1186/1749-7922-5-16

**Published:** 2010-06-10

**Authors:** Seema Biswas, Maria Vedanayagam, Gabrielle Hipkins, Andrew Leather

**Affiliations:** 1 Department of Surgery, Kings College Hospital, Denmark Hill, SE5 9RS, London

## Abstract

We report a case of traumatic inguinal hernia following blunt abdominal trauma after a road traffic accident and describe the circumstances and technique of repair. The patient suffered multiple upper limb fractures and developed acute swelling of the right groin and scrotum. CT scan confirmed the acute formation of a traumatic inguinal hernia. Surgical repair was deferred until resolution of the acute swelling and subcutaneous haematoma. The indication for surgery was the potential for visceral strangulation or ischaemia with the patient describing discomfort on coughing. At surgery there was complete obliteration of the inguinal canal with bowel and omentum lying immediately beneath the attenuated external oblique aponeurosis. A modified prolene mesh hernia repair was performed after reconstructing the inguinal ligament and canal in layers.

To our knowledge, this is the first documented case of the formation of an acute direct inguinal hernia caused as a result of blunt abdominal trauma with complete disruption of the inguinal canal. Surgical repair outlines the principles of restoration of normal anatomy in a patient who is physiologically recovered from the acute trauma and whose anatomy is distorted as a result of his injuries.

## Background

Blunt abdominal trauma may cause both crush and shearing effects on healthy abdominal wall and viscera [1]. Acute onset indirect inguinal hernia with testicular dislocation after blunt trauma is rarely reported [2], but, to our knowledge, a case resulting in complete obliteration of the inguinal canal with direct herniation of the abdominal viscera has not been documented.

The inguinal canal extends from the anterior superior iliac spine to the pubic tubercle. A defect in the posterior wall results in a direct hernia. In our case, all boundaries of the inguinal canal including the floor, posterior, inferior, medial walls and deep and superficial rings were obliterated causing traumatic herniation of the terminal ileum and caecum beneath an attenuated external oblique aponeurosis.

We describe the timely reconstruction of the abdominal wall in the inguinal region and the importance of the restoration of normal anatomy with definitive repair after resolution of swelling and haematoma.

## Case Presentation

A 24 year old man was admitted to hospital following a road traffic accident after his motorcycle collided with a lorry. The speed of collision was 35 mph and abdominal injuries were sustained as a result of impact against the motorcycle handle bars.

On arrival to the Emergency Department the patient was haemodynamically stable and fully conscious. Primary survey revealed a soft abdomen with tenderness, swelling and bruising in right groin and scrotum. There was no previous history of groin hernia.

Secondary survey, plain X ray and CT scan confirmed a fracture dislocation of the right shoulder, open fracture of right radius and ulna, multiple right lung contusions and a new right inguinal hernia. Internal fixation of the upper limb injuries was performed.

**Figure 1:**
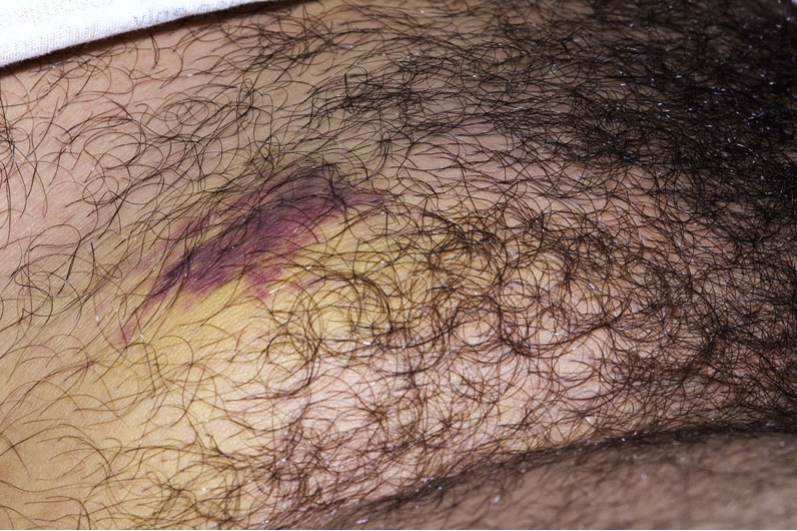
**Acute onset right groin hernia with bruising and swelling**.

Reconstruction of the abdominal wall was deferred, in the absence of obvious visceral damage, until resolution of groin swelling and bruising (Fig. 1).

**Figure 2:**
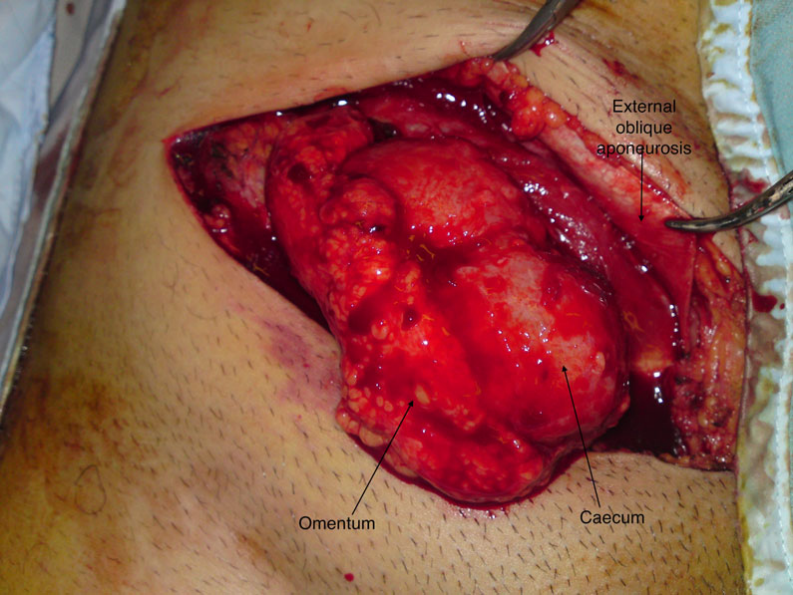
**Ileum, caecum and appendix lying immediately beneath the divided external oblique aponeurosis**.

**Figure 3:**
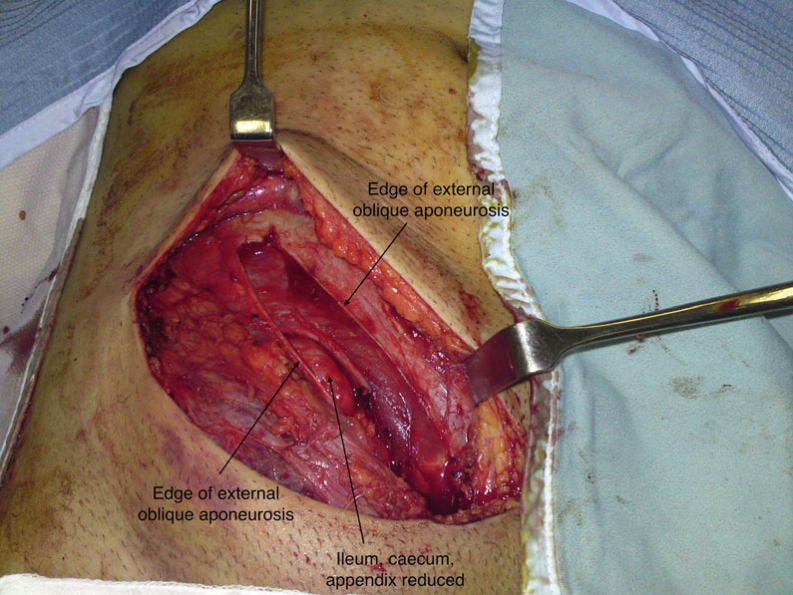
**Ileum, caecum and appendix reduced**.

**Figure 4:**
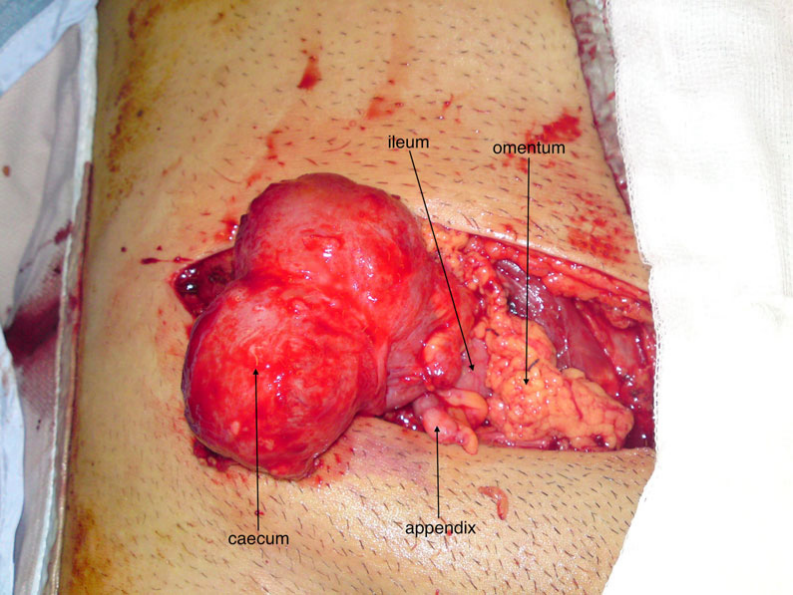
**Ileum, caecum, appendix and omentum**.

12 days after admission, repair of the inguinal hernia was performed. At surgery, the external oblique aponeurosis overlying the inguinal canal was contused inferiorly, and the inguinal ligament was found to be sheared off the full length of its attachment from the anterior superior iliac spine to the pubic tubercle, with all boundaries of the canal obliterated (Fig. 2 &3). As a result, instead of dividing the external oblique aponeurosis over the inguinal canal, as in a standard hernia repair, division was performed approximately 10 cm above the level of the inguinal canal where the fibres were intact and there was less contusion and underlying swelling. This revealed the caecum, terminal ileum, appendix and omentum lying directly beneath the external oblique aponeurosis (Fig. 4). There was no visceral ischaemia or perforation. A standard incision over the inguinal canal would, therefore, have been hazardous. Medially, the femoral artery, vein and spermatic cord were all intact and lying freely in the groin, uncontained.

**Figure 5:**
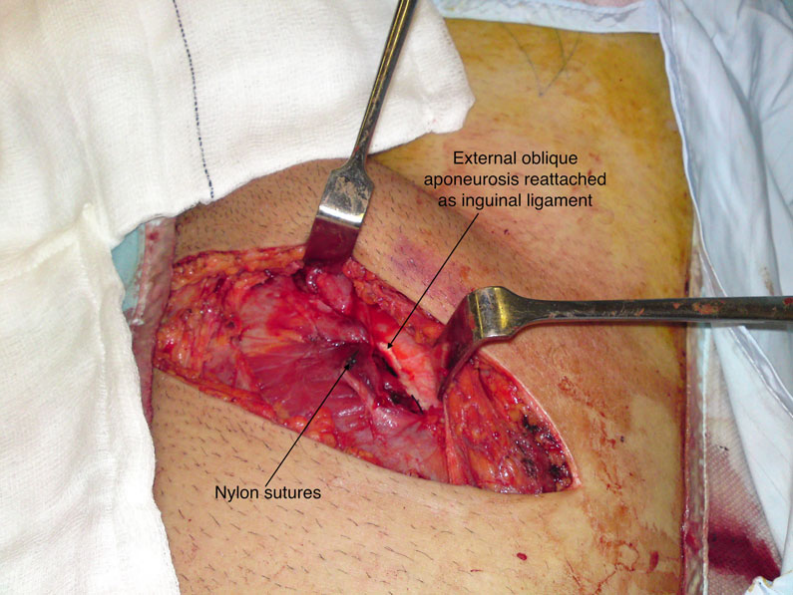
**Reconstruction of the inguinal ligament**.

**Figure 6:**
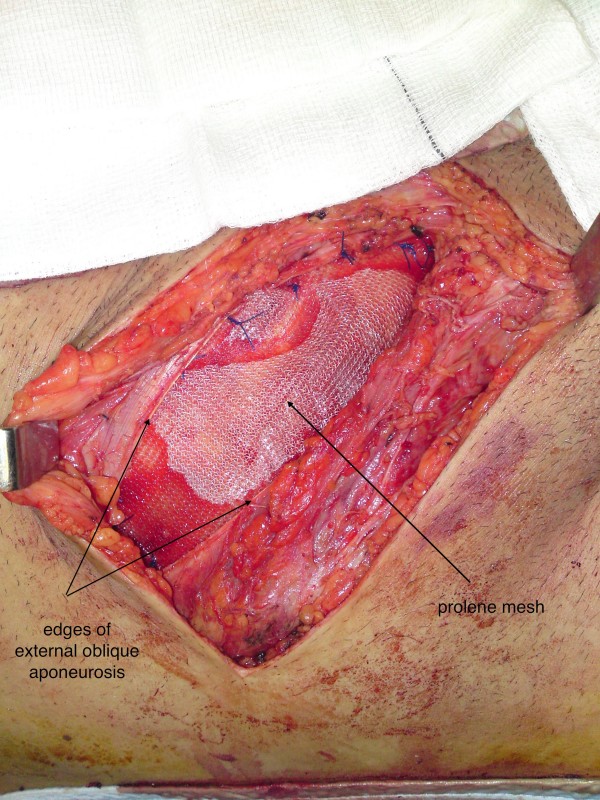
**Prolene mesh placement**.

**Figure 7:**
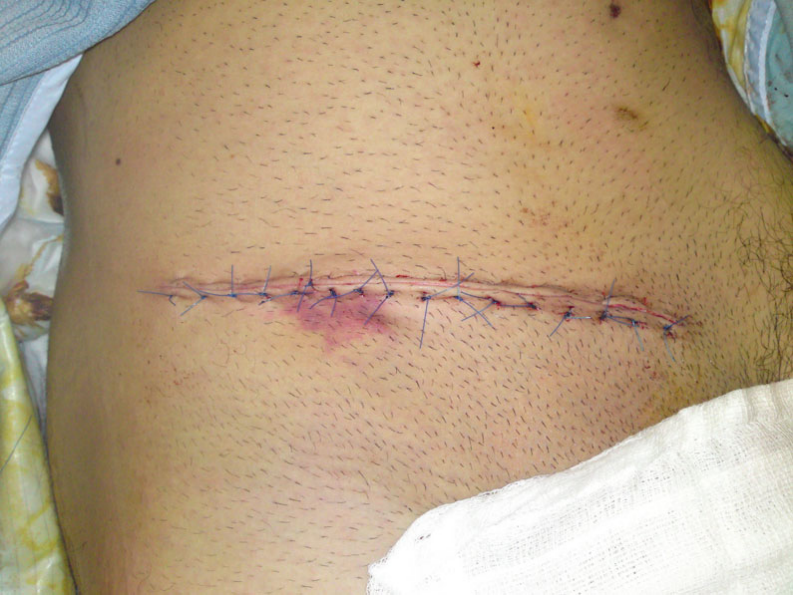
**Skin closure**.

The edge of the peritoneum was sutured to the lacunar and pectineal ligaments and pectineal line. The overlying external oblique aponeurosis was re-attached as the inguinal ligament (Fig. 5). A large piece of prolene mesh extending from the anterior superior iliac spine to the pubic tubercle was then sutured beneath the external oblique aponeurosis (Fig. 6). The external oblique was closed and skin closure achieved in layers (Fig. 7). Post-operatively the patient received antibiotics for 5 days, made an uneventful recovery and was discharged within 12 days of the initial injury. At outpatient follow-up 6 months later there were no complications.

## Conclusions

Here we discuss the first reported case of the formation and successful repair of an acute direct inguinal hernia resulting from blunt abdominal trauma where the inguinal canal was completely obliterated causing bowel to lie immediately beneath an attenuated external oblique aponeurosis. Technically there was no direct or indirect hernia as there was no inguinal canal. Traumatic injuries do not respect abdominal planes; normal anatomy is frequently distorted. Delayed repair afforded the resolution of haematoma and oedema that may have resulted in more challenging surgery.

As the defect was unilateral and the procedure was exploratory in the first instance an open approach was undertaken. The size of the defect afforded easy inspection of the peritoneal cavity for visceral injury. As primary repair was feasible without tension this was undertaken by reconstructing the inguinal region in layers. An alternative technique of repair would have been a laparoscopic intraperitoneal approach rather than extraperitoneal due to the location of abdominal viscera beneath the skin and obliteration of the abdominal wall in the right inguinal region. After reduction of the abdominal viscera composite mesh would be fixed to edges of the defect rather than direct suture of the cranial and caudal borders of the defect (edge of abdominal wall lined by peritoneum and pubic bone, respectively) together.

## Consent

Written informed consent was obtained from the patient for publication of this case report and any accompanying images. A copy of the written consent is available for review by the Editor-in-Chief of this journal.

## Competing interests

The authors declare that they have no competing interests.

## Authors' contributions

**SB **carried out the operation detailed in this report and drafted the case presentation section of the report. **MV **and **GH **drafted and compiled the document. **AL **gave approval of the manuscript before publishing. All of the above authors were involved in the care of the patient whilst in hospital.
